# Train like an athlete: applying exercise interventions to manage type 2 diabetes

**DOI:** 10.1007/s00125-020-05166-9

**Published:** 2020-06-11

**Authors:** Mladen Savikj, Juleen R. Zierath

**Affiliations:** 1 Department of Molecular Medicine and Surgery, Integrative Physiology, Karolinska Institutet, 171 77 Stockholm, Sweden; 2 Department of Physiology and Pharmacology, Integrative Physiology, Karolinska Institutet, Stockholm, Sweden; 3 Novo Nordisk Foundation Center for Basic Metabolic Research, Faculty of Health and Medical Sciences, University of Copenhagen, Copenhagen, Denmark

**Keywords:** Aerobic training, Blood glucose, Exercise, Resistance training, Review, Training intensity, Type 2 diabetes

## Abstract

Exercise elicits high energy demands, stimulating cardiorespiratory function and substrate mobilisation and oxidation. Repeated bouts of exercise lead to whole-body adaptations, which improve athletic performance. Distinct exercise modalities and intensities and nutritional conditions pose specific physiological challenges, subsequently inducing different adaptations to training. Athletes often modify these variables to achieve individualised training goals and maximise performance. Exercise training improves glycaemic control in individuals with type 2 diabetes; however, the precise training regimen that confers the most beneficial metabolic adaptations in this population is unknown. In this review, we discuss how modifying exercise type, intensity and modality and nutritional status affects the beneficial effects of exercise on glycaemic control in individuals with type 2 diabetes. Evidence indicates that greater improvements in glycaemic control can be achieved through combined aerobic and resistance training regimens compared with either training type alone. However, the increased frequency of training and a greater number of exercise bouts during combined programmes could be responsible for apparent advantages over a single training modality. The beneficial effects of aerobic exercise on glycaemic control seem to rise with training intensity, with superior adaptations achieved by high-intensity interval training (HIT). In addition, training with low carbohydrate availability (‘training low’) improves cardiorespiratory function and skeletal muscle oxidative capacity more than conventional training in healthy untrained individuals. Examinations of various training regimens are warranted to assess the safety, efficacy, feasibility and beneficial effects in the type 2 diabetes population. Just like competitive athletes, individuals with type 2 diabetes should be encouraged to adopt training regimens that improve fitness and metabolism.

**Electronic supplementary material:**

The online version of this article (10.1007/s00125-020-05166-9) contains a slideset of the figures for download, which is available to authorised users.

**Figure:**
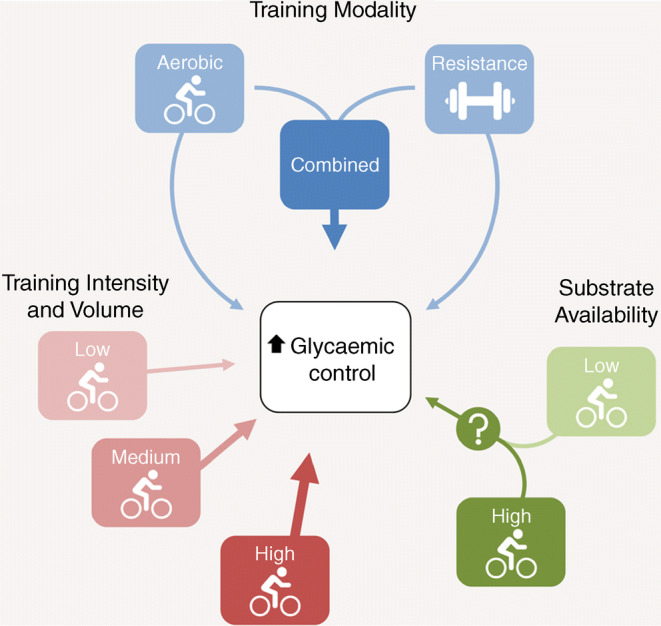
Graphical abstract

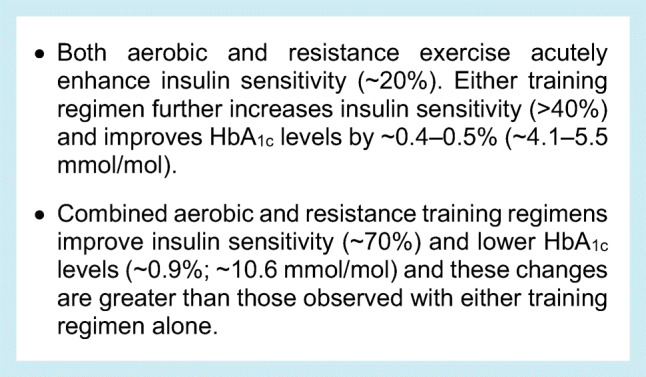

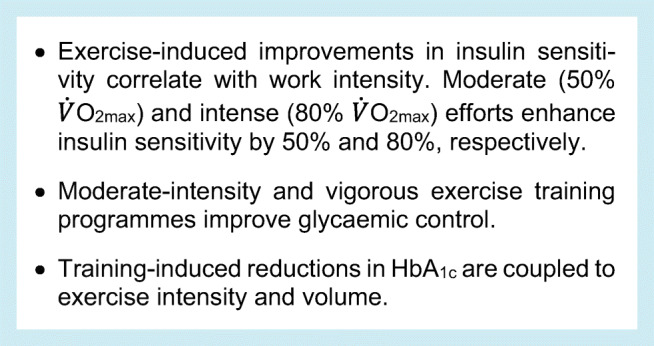

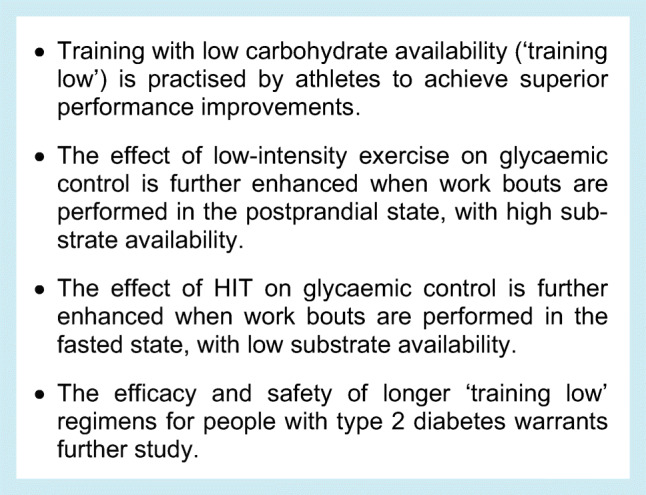







Exercise elicits a state of high energy demand, stimulating numerous bodily functions, which then work in concert to maintain energetic homeostasis. Cardiorespiratory function and substrate mobilisation and oxidation rise to meet this challenge (Fig. 1). Respiration accelerates and deepens (frequency >40 breaths/min and tidal volume ~3–4 l), increasing effective lung ventilation 20-fold (~200 l/min) during intense exercise [1]. Concurrently, cardiac output surges (20–40 l/min) due to accelerated heart rate and stroke volume (frequency up to 200 beats/min and 60–100% increase in volume) [1]. Oxygen- and substrate-rich blood is diverted to active musculature by changes in vascular conductance and the action of the skeletal muscle blood pump [1]. Such stimulation of cardiorespiratory function effectively raises oxygen and substrate delivery to skeletal muscle, providing the means for higher fuel oxidation. The adaptations that occur in response to each exercise bout are quite remarkable, and clinical physiologists have used this knowledge to apply training regimens to improve human health.

**Fig. 1:**
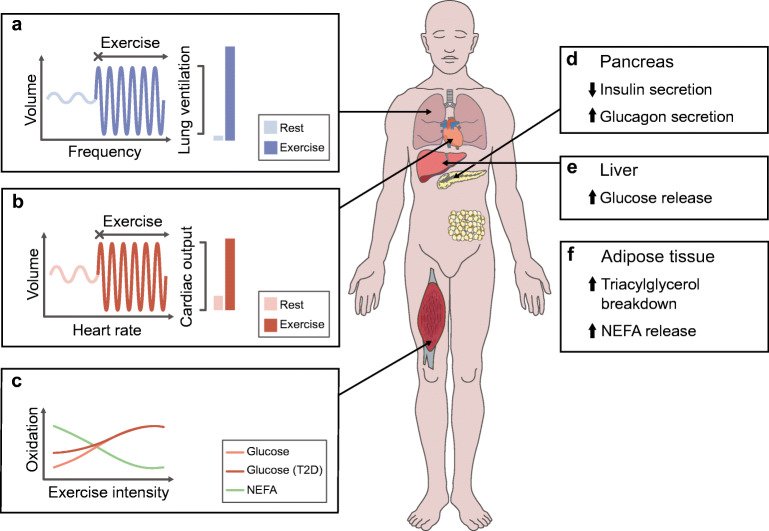
Acute response to an exercise bout. (**a**) Once exercise commences, respiratory frequency and tidal volume rise, increasing lung ventilation 20-fold compared with rest. (**b**) Similarly, increased heart rate and stroke volume lead to a fivefold higher cardiac output. Increased cardiorespiratory function allows for greater substrate and oxygen delivery to the active skeletal muscle. (**c**) NEFA are preferentially oxidised by skeletal muscle during low-intensity exercise, whereas glucose is the preferred fuel source during high-intensity exercise. Individuals with type 2 diabetes with hyperglycaemia show a greater reliance on glucose oxidation during lower-intensity exercise compared with healthy control individuals. (**d–f**) High substrate availability is maintained through glucose and NEFA release by the liver and adipose tissue, respectively, supported by increased glucagon and decreased insulin secretion by the pancreas. T2D, type 2 diabetes. This figure is available as part of a downloadable slideset

Substrate utilisation preference depends on exercise modality, intensity and duration and the nutritional status of the individual. Skeletal muscle relies on glycogen stores and exogenous substrates (blood glucose and non-esterified fatty acids; NEFA) to meet the energetic needs. The relative contribution of glycogen is greater during the initial stages of exercise, while reliance on exogenous substrates rises as the work bout persists [2]. Skeletal muscle glucose uptake rises ~7-fold during low-intensity and ~20-fold during higher-intensity exercise [3]. Concurrently, the contribution of blood glucose to substrate oxidation increases from ~10% initially, to ~30% during low-intensity and ~40% during high-intensity exercise [3]. NEFA can account for up to 50% of substrate oxidation during low-intensity exercise [2]. Hence, glucose is the preferred fuel source during high-intensity exercise, while NEFA are predominantly utilised during low-intensity exercise. The dependency on glucose oxidation during low-intensity exercise is higher in individuals with type 2 diabetes presenting with hyperglycaemia, with a lower reliance on glycogen [4]. Increased glucose availability is likely to play a role in this shift, as mild glycogen sparing occurs in healthy individuals when exercising during a constant glucose infusion [5]. Hence, this shift towards glucose preference is likely to be relevant only in hyperglycaemic individuals. The relative contribution of exogenous substrates is further modulated by nutritional status. Consuming a high-carbohydrate meal before exercise promotes greater glucose utilisation than a low-carbohydrate meal [6, 7]. Conversely, NEFA are the predominant fuel source when exercising under fasted conditions [7]. Exercise suppresses insulin release and promotes glucagon secretion, maintaining elevated blood glucose through hepatic glucose release, while adrenergic stimulation maintains elevated NEFA levels by stimulating adipose tissue triacylglycerol breakdown [1]. Hence, external substrate utilisation preference depends on the exercise modality and the timing of the training bout and is supported by substrate mobilisation in the adipose tissue and liver.

## Expanding performance limits through training

**Fig. 2:**
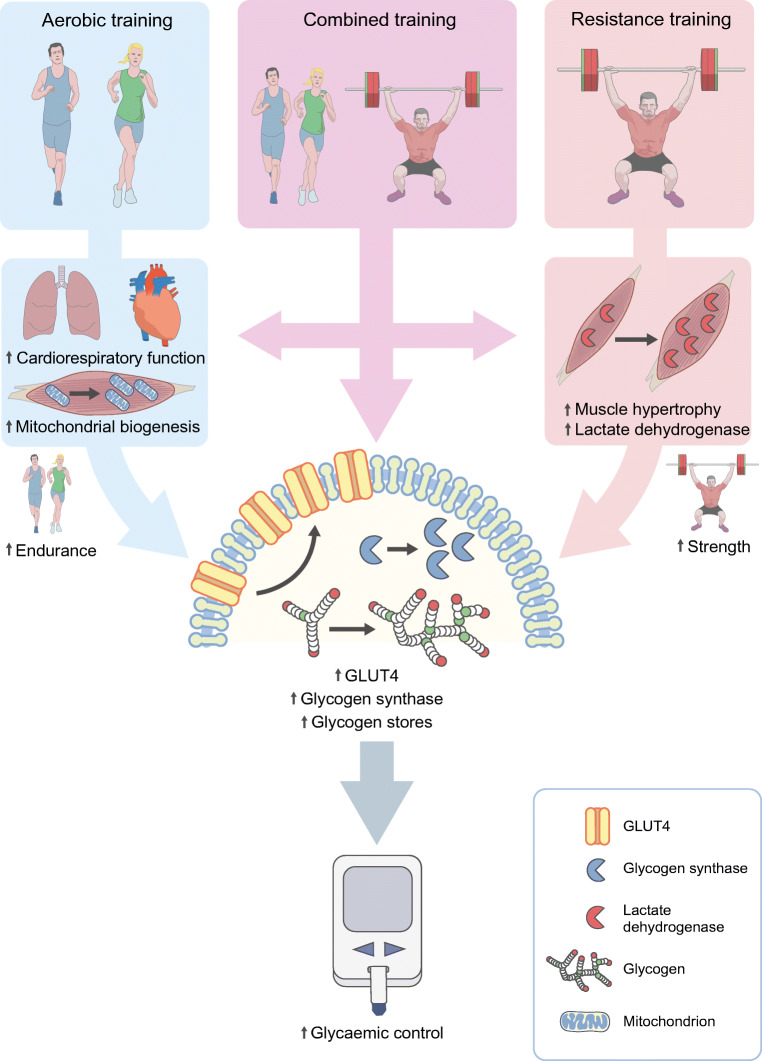
Adaptations to different training types. Aerobic training enhances cardiovascular function and promotes skeletal muscle mitochondrial biogenesis, thereby improving exercise endurance. Resistance training promotes skeletal muscle hypertrophy and increases strength, allowing for more powerful contractions fuelled by glycolysis and supported by higher lactate dehydrogenase content. A combined aerobic and resistance training programme improves endurance and strength/power, albeit to a lesser extent than individual forms of training. However, all training types improve skeletal muscle glucose transport and glycogen synthesis capacity, expanding glycogen stores and improving glycaemic control. This figure is available as part of a downloadable slideset

During training, repeated homeostatic challenges induce cardiorespiratory and skeletal muscle adaptations, which improve athletic performance. Distinct training types (aerobic or resistance) pose specific physiological challenges and induce different adaptations (Fig. 2). Resistance training promotes muscle fibre growth and transformation to a glycolytic phenotype, with increased percentage of fast-twitch fibres and lactate dehydrogenase content, while aerobic training increases mitochondrial density, oxidative enzymes and the relative proportion of slow-twitch fibres [2]. Partly as a result of focused resistance training, elite shot-putters have skeletal muscle composed predominantly (~60%) of large, fast-twitch fibres suited to explosive, glycolysis-driven actions, such as propelling ~7.25 kg shot-puts over 20 m [8]. Conversely, the skeletal muscle of distance runners contains predominantly (~70%) slow-twitch fibres and has a high mitochondrial content, allowing prolonged efforts supported by substrate oxidation [8]. Additional cardiorespiratory adaptations allow elite athletes to reach remarkable maximal oxygen consumption rates ($\dot{V}{\mathrm{O}}_{2\max }$; ~80 ml kg^−1^ min^−1^) compared with untrained individuals (~40 ml kg^−1^ min^−1^) and endure exceptional workloads [1]. Combined training improves strength and endurance to a lesser extent than focused regimens of either resistance or endurance, respectively [9]. While elite athletic performance is truly remarkable, training adaptations in the general population are more discreet, with fibre-type composition and $\dot{V}{\mathrm{O}}_{2\max }$ showing modest improvements [10, 11]. Variations in such improvements are likely to be caused by genetic differences, as training-induced improvements in $\dot{V}{\mathrm{O}}_{2\max }$ show notable familial distribution [10]. However, even modest improvements in strength and conditioning are clinically meaningful, as low fitness levels are a relevant predictor of mortality [12]. While an athlete’s choice of training depends on performance goals, the primary measure of success of a training regimen for the management of type 2 diabetes is the extent of improvement in glycaemic control, which will be the focus of this review.

## Metabolic benefits of different training types

Exercise increases skeletal muscle glucose uptake by an insulin-independent mechanism [13]. Unsurprisingly, canonical acute responses to exercise, such as increased AMP-activated protein kinase activity and expression of the gene encoding peroxisome proliferator-activated receptor γ coactivator 1α (*PGC-1α*, also known as *PPARGC1A*) are conserved in insulin-resistant skeletal muscle [14]. Exercise training also restores skeletal muscle mitochondrial function in individuals with type 2 diabetes and induces gains in strength and endurance that are comparable to those observed in healthy individuals [15]. Although insulin sensitivity is also improved through exercise training, it remains lower than that in healthy control individuals [16]. The age of the participant or duration of type 2 diabetes may influence the metabolic adaptations to exercise training, with younger individuals showing greater improvements in metabolic control [17]. Nevertheless, exercise type, intensity and volume and training duration can be modulated to achieve glycaemic improvements [18]. While a complete remission of insulin resistance may not always be achieved, better glycaemic control can be attained by fine-tuning the training modality.

Participation in any type of training regimen improves glycaemic control [19], but the relative advantage conferred by specific exercise types is less clear. Aerobic training has traditionally been prescribed for type 2 diabetes management, supported by evidence from RCTs showing lower (by 0.5–3.1%; 5.5–33.4 mmol/mol) HbA_1c_ levels after 2–6 months of moderate- to high-intensity aerobic training as compared with conventional treatment [20–23]. A meta-analysis of nine RCTs reported a small but consistent and relevant decrease in HbA_1c_ levels of 0.67% (~7.5 mmol/mol) in response to aerobic training in individuals with type 2 diabetes [19]. Such beneficial effects on glycaemic control are due to two essential characteristics of aerobic exercise, namely to bypass insulin resistance and increase skeletal muscle glucose uptake and to potentiate insulin sensitivity following an exercise bout [24]. Acute aerobic exercise increases whole-body insulin sensitivity, enhancing skeletal muscle glucose uptake, and this effect persists for >48 h, while exercise training confers further improvements [2]. The response to both high- and low-dose insulin infusion during a hyperinsulinaemic clamp improves after training (45% with 0.24 pmol m^−2^ min^−1^ and 17% with 6 pmol m^−2^ min^−1^ insulin infusion), indicating increased insulin sensitivity and responsiveness [25]. Exercise-induced improvements in insulin action are likely to result from skeletal muscle adaptations, such as increased glycogen stores and synthesis rates, supported by increases in GLUT4 protein content and glycogen synthase activity [1, 2]. However, improvements acquired through aerobic training may be temporary as fasting glucose and insulin values regress to pre-training levels ~72 h after the last training bout [26]. Thus, while aerobic training improves insulin sensitivity and glycaemic control, maintaining the exercise regimen may be necessary to preserve these benefits.

A single bout of resistance exercise leads to an acute improvement in glycaemic control, and decreased fasting glucose and insulin levels (by 0.2 mmol/l and 17.4 pmol/l vs pre-training, respectively) the subsequent morning [27]. RCTs show that longer resistance training periods (5–6 months) lead to lower HbA_1c_ levels (0.4–0.8%, 4.1–8.7 mmol/mol) than those observed with standard care in the type 2 diabetes population [23, 28]. These improvements are likely to be due to skeletal muscle adaptations, as 6 weeks of unilateral lower-limb resistance training increases glucose uptake (18%), GLUT4 protein content, and glycogen synthase activity in the trained vs untrained skeletal muscle of individuals with type 2 diabetes [29]. Controlled trials of 4–6 weeks of extensive whole-body training show heightened insulin sensitivity and increased glucose disposal (48%) during a hyperinsulinaemic clamp at least 48 h after the last resistance exercise bout [30]. Hence, in addition to aerobic regimens, resistance training regimens are efficacious in improving glycaemic control in individuals with type 2 diabetes.

The relative efficacy of an aerobic, resistance or combined training regimen for improving glycaemic control has been assessed in individuals with type 2 diabetes. An RCT of a 6 month training regimen (aerobic, resistance or both combined) showed that HbA_1c_ levels are 0.4–0.9% (4.1–10.6 mmol/mol) lower after any training regimen compared with standard care [23]. A parallel 4 month trial of aerobic or resistance training reported similar reductions in HbA_1c_ levels (~0.4%; ~4.4 mmol/mol), with mild advantage of aerobic over resistance training on improvements in insulin sensitivity (30% vs 15% improvement) [31]. However, a combination of aerobic and resistance training reduces HbA_1c_ levels to a greater extent (0.9%; 10.6 mmol/mol) than either regimen alone (0.4–0.5%; 4.1–5.5 mmol/mol) [23]. Similarly, an RCT showed greater insulin-sensitising effects of 4 months of combined training compared with aerobic training alone, as evidenced by greater glucose disappearance rates during a hyperinsulinaemic clamp (77% and 20% increase for combined and aerobic training, respectively) [32]. These differences may partly be accounted for by larger increases in skeletal muscle mass and subsequent expansion of glycogen storage capacity caused by the resistance exercise component [32]. However, in such reports, normalising the volume and workload of essentially different exercise types can prove difficult. Attempts at normalising were made in this 4 month study by estimating the energy expenditure associated with each exercise component [32], while in other studies no normalisation is performed and the volume of combined training is larger than either training regimen alone [23]. Thus, although there are indications of superior glycaemic improvements with combined training, larger exercise volumes may account for some of these changes. Regardless, training of any type improves glycaemic control and should be encouraged, even if the specific training type is deferred to personal choice.

## Effects of training volume and intensity on metabolic adaptations

**Fig. 3:**
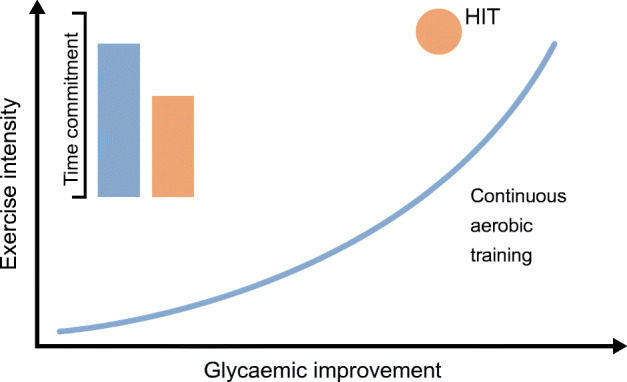
Glycaemic effects of different training intensity. Beneficial effects of continuous aerobic exercise (blue line) on glycaemic control with exercise intensity. HIT (orange circle) confers superior glycaemic improvement as compared with continuous moderate-intensity training, with a lower time commitment (1.5 vs 2.5 h/week; inset bars). This figure is available as part of a downloadable slideset

Intensity and volume are important factors to consider when initiating a training regimen specifically pertaining to aerobic exercise. An RCT examined the effects of 6 months of aerobic training at different intensities (moderate or vigorous, 40–55% or 65–85% peak oxygen consumption [$\dot{V}{\mathrm{O}}_{2\mathrm{peak}}$], respectively) and volumes (low volume ~5000 kJ/week or high volume ~8350 kJ/week) on metabolic variables in overweight, sedentary individuals [33]. Insulin sensitivity, measured during an IVGTT ~24 h after the final training bout, increased with training (25–65% vs pre-training), regardless of intensity or volume [33]. However, examination of the same study group showed that, 15 days after the last training bout, insulin sensitivity remained higher only in the moderate, low-volume and vigorous, high-volume aerobic training groups (~15% vs pre-training) [34]. These differences may be accounted for by the greater expansion of glycogen stores and mitochondrial capacity of skeletal muscle in the vigorous, high-volume group, and by longer and more frequent exercise sessions in the moderate, low-volume and vigorous, high-volume groups [34]. In individuals with impaired glucose tolerance, the acute insulin-sensitising effect is stronger after a vigorous bout of exercise than after an isoenergetic, moderate bout of exercise (exercise at ~80% and ~50% $\dot{V}{\mathrm{O}}_{2\max }$ improved insulin sensitivity by 80% and 50%, respectively) [35]. However, a parallel trial of 6 months of vigorous and moderate-intensity (75% and 50% $\dot{V}{\mathrm{O}}_{2\mathrm{peak}}$, respectively) aerobic training of low- or high-volume (42 and 67 kJ kg^−1^ week^−1^, respectively) exercise reported similar improvements in indices of insulin sensitivity in individuals with impaired fasting glucose for all training regimens [36]. Thus, while higher exercise intensity may confer greater acute insulin sensitisation in population with elevated fasting glucose, effects of longer training may be similar, irrespective of exercise volume and intensity. Several meta-analyses of controlled clinical trials have shown a significant association between improvements in HbA_1c_ levels and exercise intensity, and a modest association with training volume in individuals with type 2 diabetes [18, 37]. While further direct examinations of this relationship are needed, higher exercise intensity and volume may be more beneficial for glycaemic control in the type 2 diabetes population (Fig. 3).

Exercise intensity can be modulated by performing an aerobic bout continuously or by alternating high-exertion and low-exertion intervals, known as high-intensity interval training (HIT). Parallel trials have shown that 6 weeks of HIT induces similar athletic performance adaptations to continuous moderate-intensity training in healthy, untrained individuals [38]. Interestingly, HIT improves performance with a lower time commitment compared with continuous moderate-intensity training (1.5 vs 4.5 h/week, respectively), making HIT an alluring alternative for the engaged contemporary lifestyle [38]. In addition, an acute HIT bout effectively lowers blood glucose levels to a similar extent as an isocaloric bout of moderate intensity [39]. An RCT has shown that 3 months of HIT lowers fasting glucose (by 0.7 mmol/l), reduces HOMA-IR (by 25%) and decreases glycaemic variability (by 5%) in individuals with type 2 diabetes [40]. These adaptations are comparable to those seen in response to continuous moderate-intensity aerobic training, despite the 40% lower time commitment (~1.5 vs 2.5 h/week) and exercise volume for HIT [40]. However, the extent to which untrained individuals can tolerate high-intensity training and their willingness to engage in such regimens are commonly raised concerns. Reports examining exercise tolerance show that untrained individuals do indeed find HIT more exhausting, but also more enjoyable, than continuous moderate-intensity exercise [41]. Thus, owing to its efficacy in improving glycaemic control with lower time commitment and training volume, HIT may be a preferable alternative to more traditional continuous aerobic exercise (Fig. 3). Further studies examining the feasibility of longer training interventions are needed to address the training tolerance and participation rate concerns for individuals with type 2 diabetes.

## Effects of substrate availability on metabolic adaptations to training

**Fig. 4:**
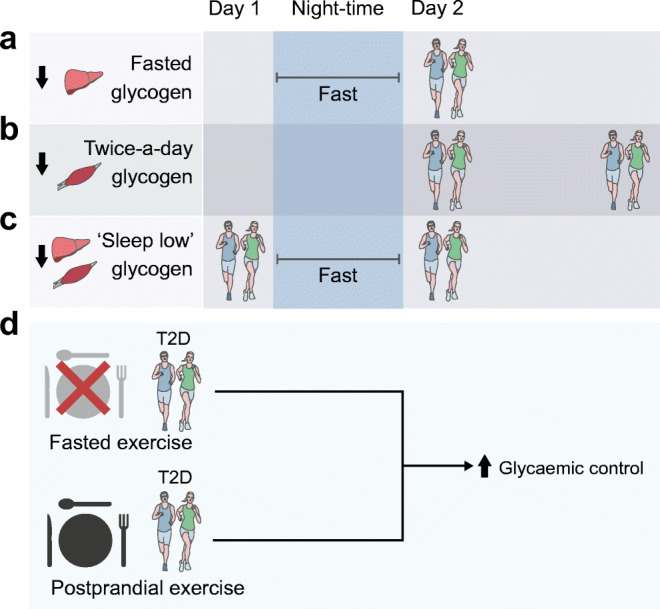
Training with low carbohydrate availability. Athletes achieve training in a low-carbohydrate state by several methods. (**a**) Fasting overnight, before an exercise bout, depletes liver glycogen content and lowers carbohydrate availability. (**b**) Training twice a day depletes skeletal muscle glycogen content during the first exercise bout, allowing the second bout to be initiated with low carbohydrate availability. (**c**) The ‘sleep low’ strategy encompasses both, by depleting skeletal muscle glycogen through an evening bout of exercise, and liver glycogen by fasting, before the second bout of exercise in the morning. (**d**) In individuals with type 2 diabetes, superior glycaemic improvements can be achieved through high-intensity exercise in postprandially or low-intensity exercise under fasted conditions. T2D, type 2 diabetes. This figure is available as part of a downloadable slideset

Fuel preference (lipid or carbohydrate) during exercise partly depends on substrate availability and confers different training adaptations to exercise. Athletes put these differences to good use, modulating food intake and training schedules to achieve superior performance. High carbohydrate availability confers the best performance during an activity bout and consumption of carbohydrate-rich beverages reduces symptoms of overtraining [42]. Low carbohydrate availability (‘training low’) forces the utilisation of lipids as a fuel source and promotes mitochondrial biogenesis and superior adaptations in skeletal muscle oxidative capacity [43, 44]. Traditional approaches of ‘training low’ are exercising after an overnight fast, with depleted liver glycogen, or exercising twice a day, depleting glycogen stores with the first training bout and initiating the second bout with low skeletal muscle glycogen content (Fig. 4). A novel approach encompasses both strategies and includes performing a HIT bout in the evening followed by an overnight fast, which depletes both skeletal muscle and liver glycogen stores before performing a second exercise bout the following morning (Fig. 4) [43]. This training modality confers superior increases in fatty acid oxidation, fatty acid transporter content and *PGC-1α* mRNA expression, as well as improving endurance performance over training with high carbohydrate availability [43, 44]. Therefore, ‘training low’ is often used to achieve the most beneficial training adaptations, while competitive events are performed with high carbohydrate availability to confer the best performance.

Direct examinations of such training strategies in individuals with type 2 diabetes are lacking. However, information on the acute glycaemic response to an exercise bout performed in different nutritional states is available from several crossover RCTs. Insulin sensitivity during an OGTT in healthy individuals is higher immediately after a moderate-intensity cycling bout in a postprandial state than in a fasted state (75% and 15% improvement, respectively) [45]. Similarly, a single bout of moderate treadmill walking suppresses high glucose excursions and reduces glycaemic variability in individuals with type 2 diabetes more effectively when performed in a postprandial state than in a fasted state [46]. Converse results were reported in a parallel trial examining two different training modalities (moderate-intensity continuous training and HIT) under different nutritional conditions (fasted and postprandial) in individuals with type 2 diabetes [47]. Glycaemic responses to meals were lower after both exercise types in the fasted state rather than postprandially (90% and 36% decrease in incremental AUC, respectively), while the most favourable effects on mean glucose concentration (1.5 mmol/l decrease), time spent in hyperglycaemia (58% lower), and fasted glucose levels (1 mmol/l decrease) were achieved by HIT under fasted conditions [47]. Hence, the glycaemic response to exercise in different nutritional states may be dependent on the modality and intensity, with a stronger impact of moderate exercise achieved in the postprandial state and superior effects of vigorous exercise attained under fasted conditions (Fig. 4). While these studies provide information regarding the acute responses to exercise in study protocols with divergent carbohydrate availability, they do not fully follow the principles of ‘training low’ paradigms. Studies examining longer training periods are needed to discern whether the adaptations achieved in the type 2 diabetes population reflect those achieved in healthy individuals, and whether these adaptations are superior to those achieved using conventional training regimens.

## Conclusion

Selection of the most efficacious training regimen for the management of type 2 diabetes is a complex issue and exercise type, intensity and volume and nutritional status should be considered. Current research indicates that combined resistance and aerobic training regimens and HIT convey superior improvements in glycaemic control [23, 40]. Concerns are often raised about acute side-effects of HIT in the diabetes population [48]. HIT has been applied in cardiovascular disease rehabilitation without increased risk of adverse events [48]. However, the cardiovascular health of the individual needs to be considered when initiating such training regimens. Specific modalities, such as ‘training low’, are commonly practised by athletes to induce superior physiological adaptations compared with conventional training [43, 44]. However, the efficacy, feasibility and overall effects of such training regimens in individuals with type 2 diabetes are understudied. Notably, the risk of hypoglycaemia could be higher with ‘training low’, especially in individuals with uncontrolled or insulin-treated type 2 diabetes. Although many exercise regimens are safe for most people, untrained individuals are advised to consult a physician to evaluate whether there are any underlying health issues that may negatively impact full participation. While substantial evidence supports the notion that exercise training improves glycaemic control, further studies are needed to examine the relative advantage of distinct training types and intensities, nutritional status and the interaction of these factors, to improve health outcomes in people with type 2 diabetes. Such investigations would provide additional insight into the application of exercise training regimens to confer the most advantageous metabolic adaptations to combat the rising tide of metabolic disease.

## Electronic supplementary material

Slideset of figures (PPTX 334 kb)

## Abbreviations

AMPK: AMP-activated protein kinase
HIT: High-intensity interval training
$\dot{V}{\mathrm{O}}_{2\max }$: Maximal oxygen consumption rate
$\dot{V}{\mathrm{O}}_{2\mathrm{peak}}$: Peak oxygen consumption rate
