# Supplementary material for: Train like an athlete: applying exercise interventions to manage type 2 diabetes
Source: Diabetologia. 2020 Jun 11;63(8):1491–9. doi: 10.1007/s00125-020-05166-9 (PMC7351814; doi:10.1007/s00125-020-05166-9)
Supplement: Supplementary file 1 — (PPTX 334 kb) [file 125_2020_5166_MOESM1_ESM.pptx]

## Slide 1
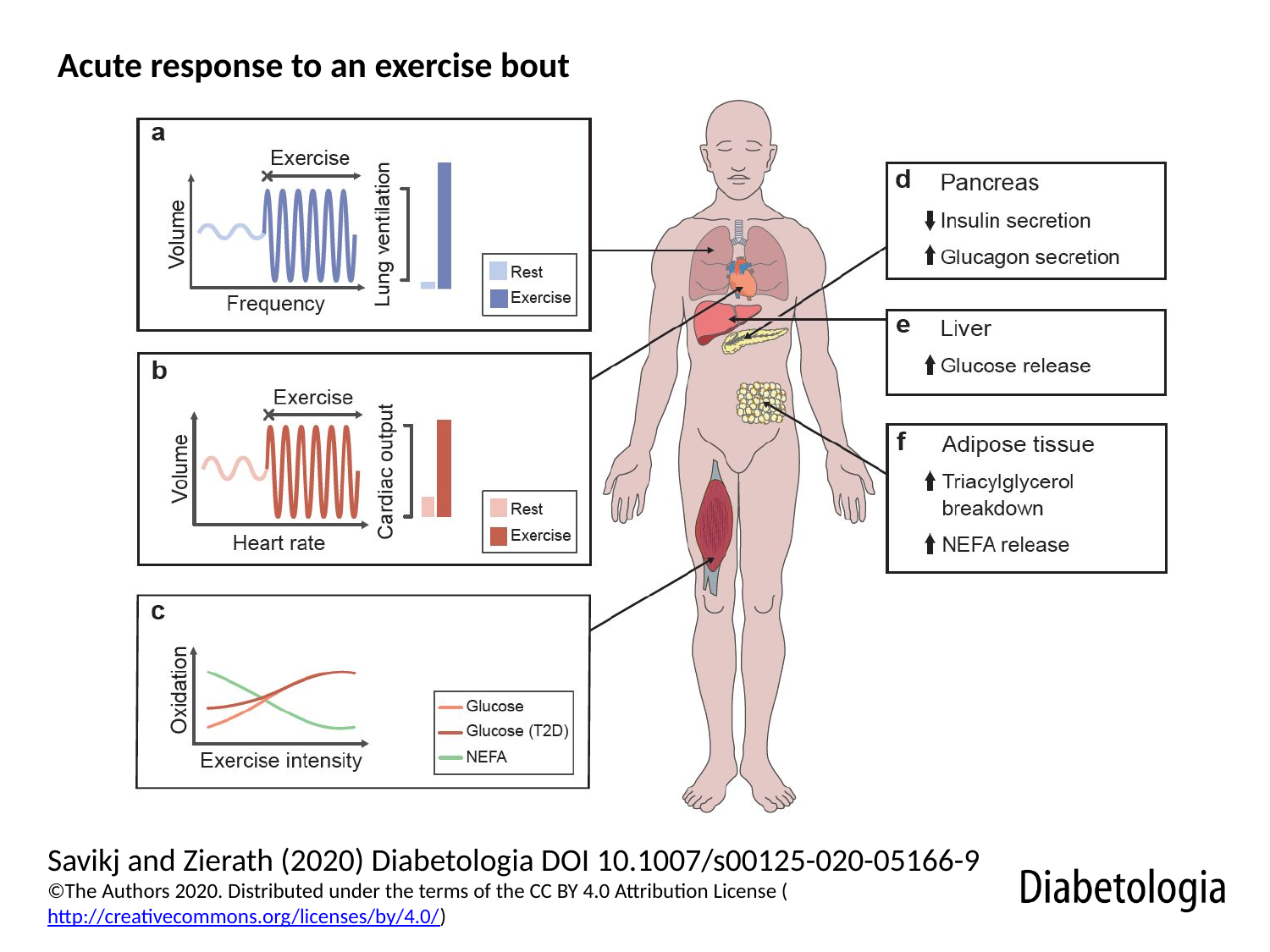

Acute response to an exercise bout
Savikj and Zierath (2020) Diabetologia DOI 10.1007/s00125-020-05166-9
©The Authors 2020. Distributed under the terms of the CC BY 4.0 Attribution License (http://creativecommons.org/licenses/by/4.0/)

## Slide 2
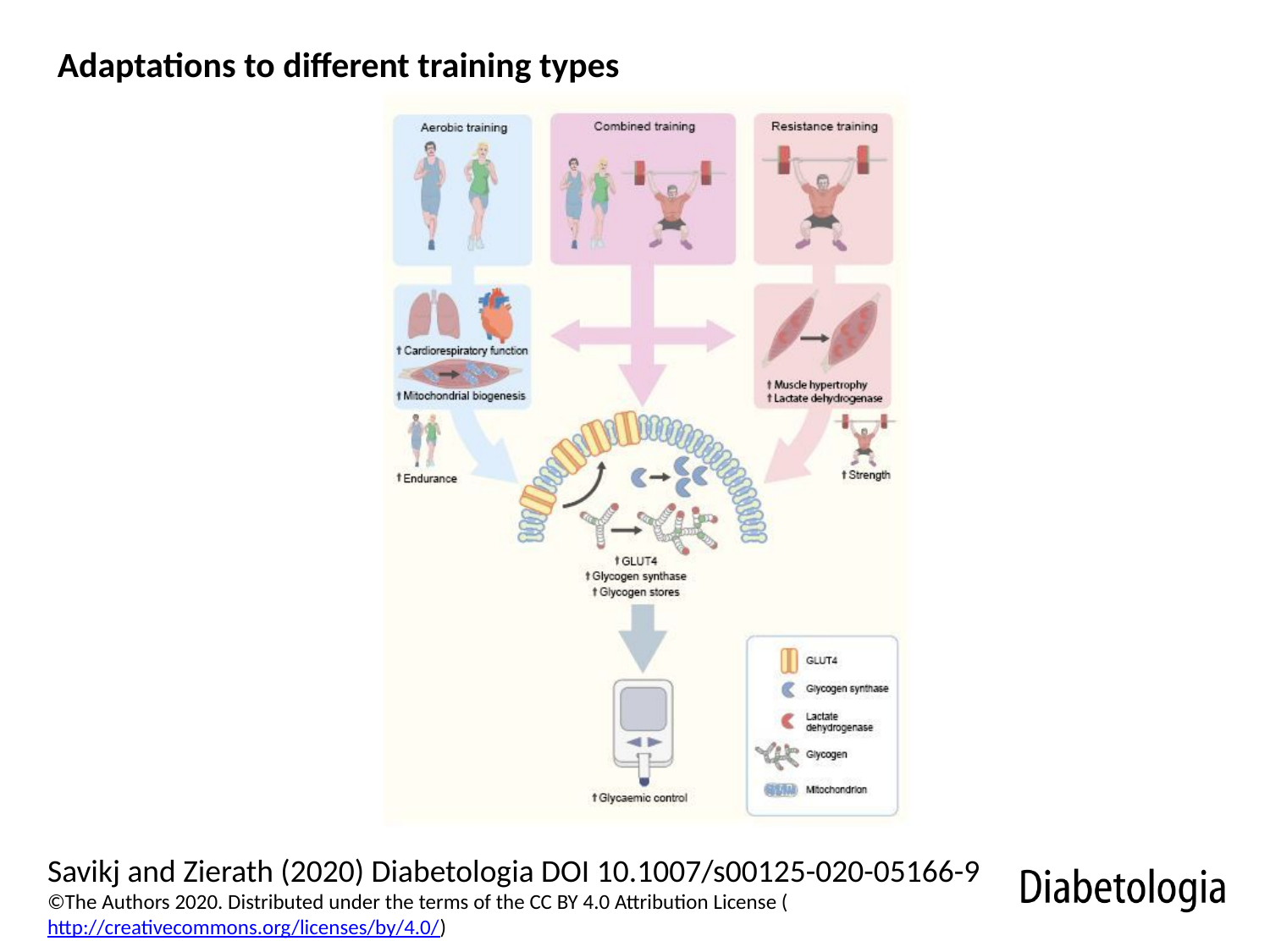

Adaptations to different training types
Savikj and Zierath (2020) Diabetologia DOI 10.1007/s00125-020-05166-9
©The Authors 2020. Distributed under the terms of the CC BY 4.0 Attribution License (http://creativecommons.org/licenses/by/4.0/)

## Slide 3
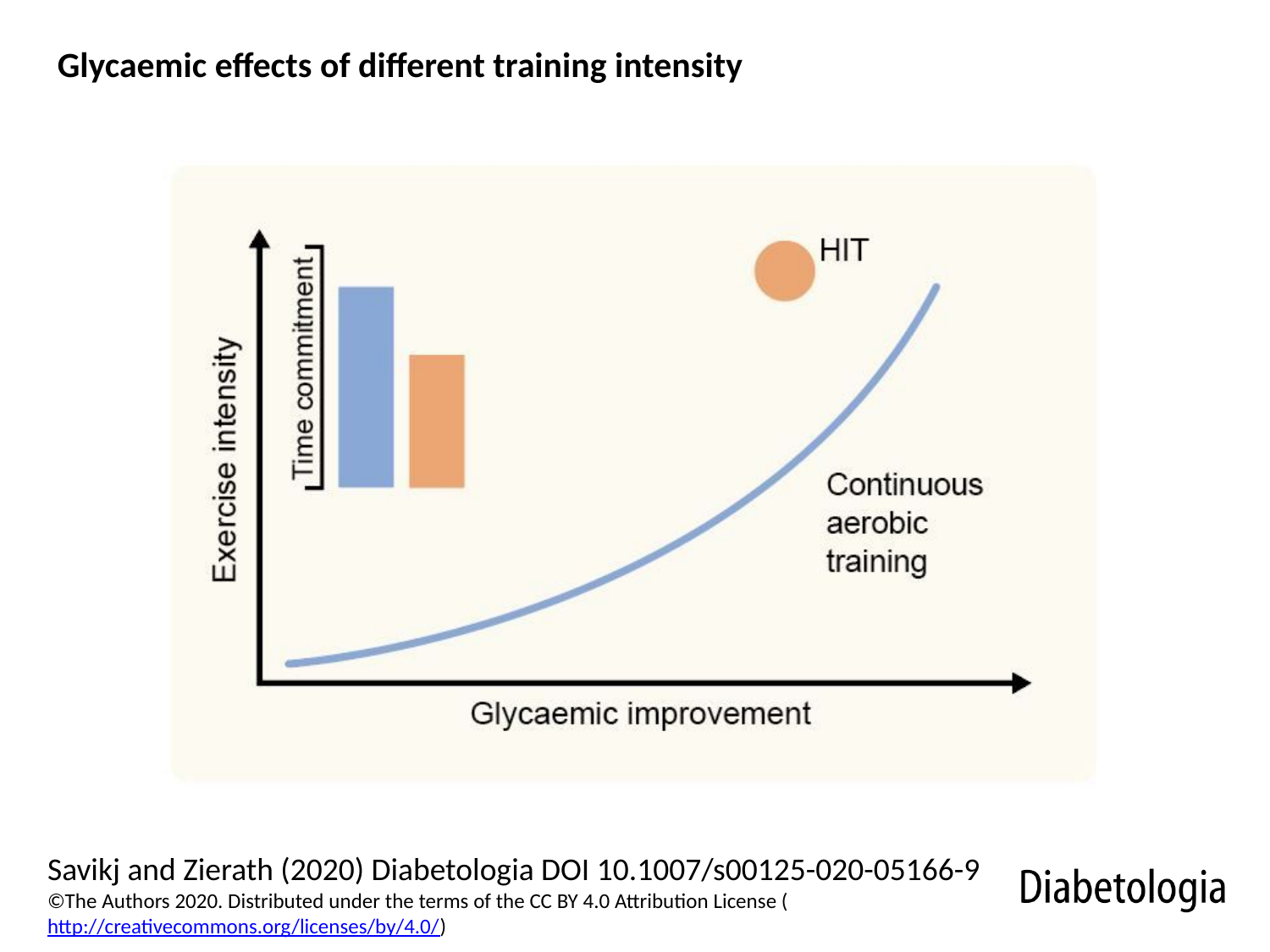

Glycaemic effects of different training intensity
Savikj and Zierath (2020) Diabetologia DOI 10.1007/s00125-020-05166-9
©The Authors 2020. Distributed under the terms of the CC BY 4.0 Attribution License (http://creativecommons.org/licenses/by/4.0/)

## Slide 4
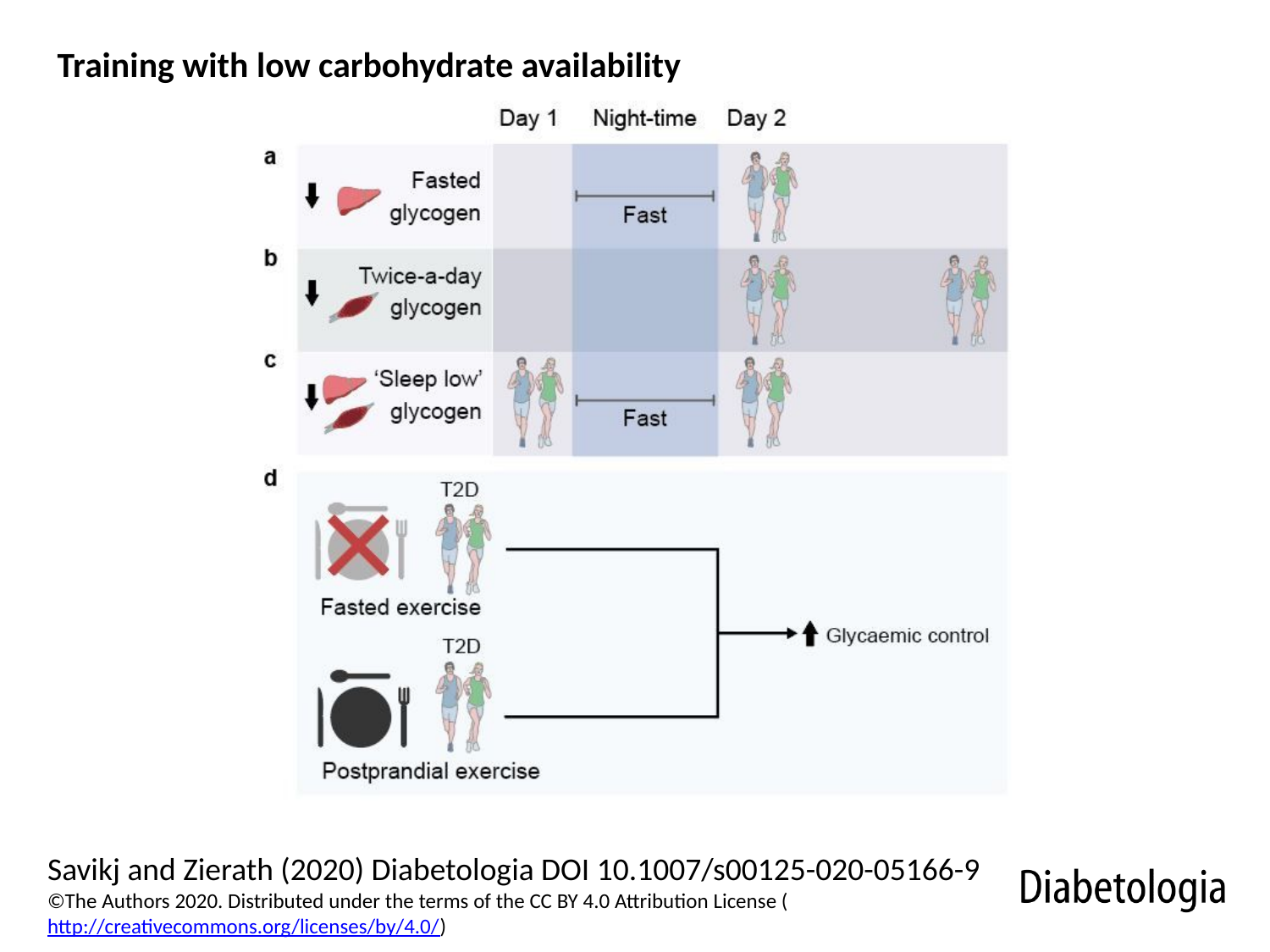

Training with low carbohydrate availability
Savikj and Zierath (2020) Diabetologia DOI 10.1007/s00125-020-05166-9
©The Authors 2020. Distributed under the terms of the CC BY 4.0 Attribution License (http://creativecommons.org/licenses/by/4.0/)
